# A Patient With Hemoptysis and the Sign of the Camalote

**DOI:** 10.1093/ofid/ofy286

**Published:** 2018-11-02

**Authors:** Alex Studemeister, Marcos N Alvarez, Lucy Studemeister

**Affiliations:** 1 Department of Medicine, Section of Infectious Diseases, El Camino Hospital, Mountain View, California; 2 Departments of Radiology and Vascular and Interventional Radiology, El Camino Hospital, Mountain View, California; 3 Ponce Health Sciences University, School of Medicine, Ponce, Puerto Rico

**Keywords:** echinococcus, hydatid cyst, sign of the camalote, water lily sign

## Abstract

A 30-year-old woman presenting with hemoptysis followed by acute respiratory distress developed a diagnostic pulmonary radiographic finding, called the sign of the camalote, indicative of ruptured hydatid cyst. Her computed tomography scan demonstrated the characteristic detached parasitic membrane floating on cystic fluid, reminiscent of Amazonian camalote leaves. She was managed successfully surgically. Ruptured hydatid cysts may present as a diagnostic challenge, in which the sign of the camalote may provide an important clue for this serious complication.

## CASE

**Figure 1.**
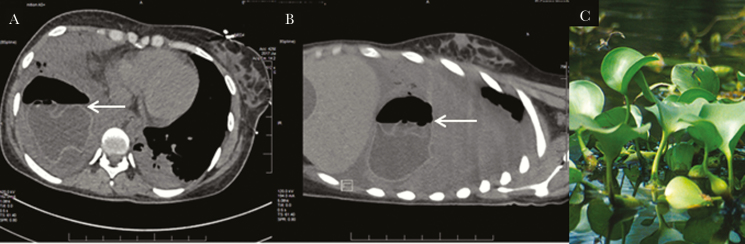
A, Computed tomography of chest, axial view, demonstrating ruptured hydatid cyst with air-fluid level and serpiginous parasitic membrane (arrow). B, Sagital view demonstrating crumpled parasitic membrane floating at the air-fluid level (arrow). C, Camalote plant (common water hyancinth), *Eichhornia crassipes*. Source: United States Department of Agriculture (www.ars.usda.gov/oc/images/photos/mar00/k8801-2, accessed August 10, 2018).

**Figure 2.**
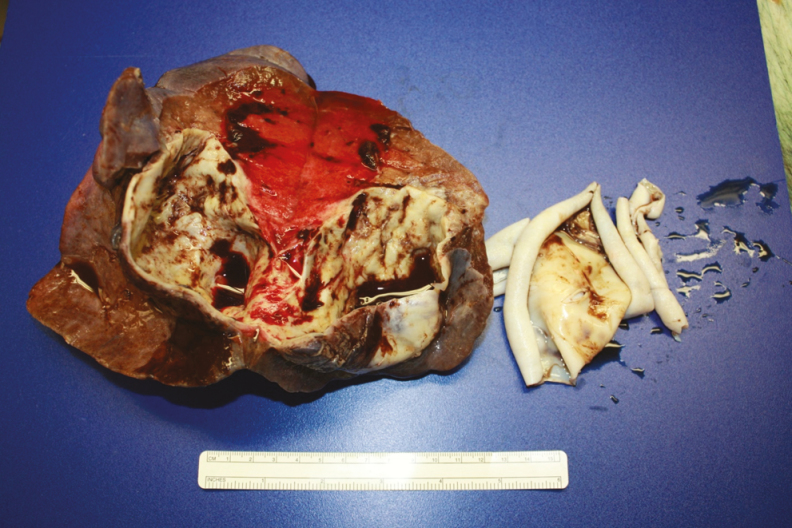
Right lower lobe of the lung, ruptured hydatid cyst, and adjacent hydatid fluid.

**Figure 3.**
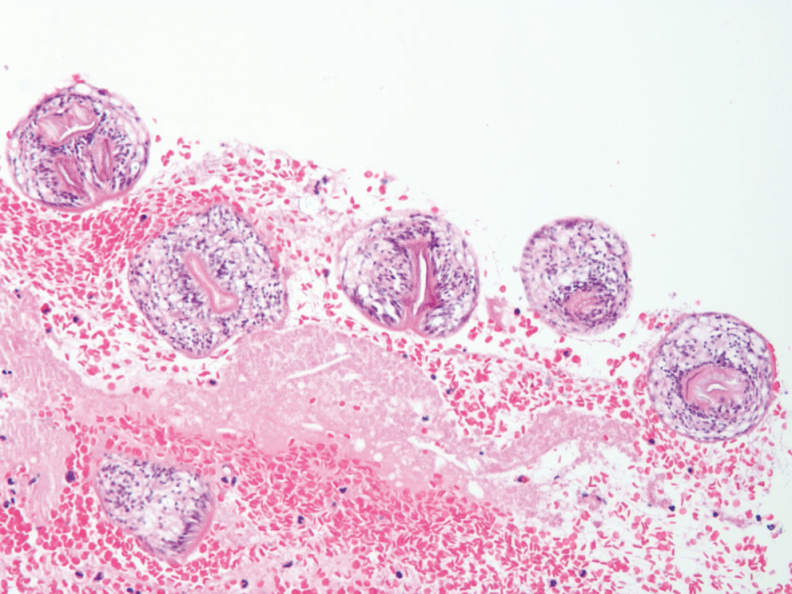
Ecchinococcal scolices from the germinal membrane of the hydatid cyst, stained with hematoxylin and eosin.

A 30-year-old woman who spent her childhood summers in northern Iran raising sheep was hospitalized with hemoptysis. Initial chest computed tomography (CT) revealed a right lower lobe cyst, 9 by 11 cm. Several days after admission, she developed respiratory distress with hypoxia. Repeat CT scan demonstrated air within the pulmonary cyst with a floating wavy membrane at the air-fluid level, indicative of the sign of the camalote (Figure 1). She underwent urgent lobectomy. Upon intubation, clear cystic fluid was suctioned from the endotracheal tube. Her right lower lobe contained a large ruptured cyst (Figure 2). Histopathology demonstrated echinococcal scolices (Figure 3). Her preoperative ecchinococcal enzyme-linked immunosorbent IgG antibody assay tested negative; it was positive postoperatively (7.15 IV, Arup Laboratories). She recovered fully and completed a 4-week course of albendazole.

## DISCUSSION

Hydatid lung disease, caused by the cestode, *Echinococcus granulosus*, commonly presents radiologically as a round density. Most persons remain asymptomatic for years until cyst perforation, mass effect, or secondary infection. As the hydatid cyst slowly enlarges, it may erode into the bronchial tree with the introduction of air into the cyst wall, causing collapse of the inner endocyst membrane. The collapsed membrane within the cyst forms a serpiginous structure within the cyst (Figure 1A). The detached floating membrane appears as a wavy structure at the air-fluid level, producing the sign of the camalote (Figure 1B). At this stage, patients may develop cough, hemoptysis, and expectoration of cyst fluid, membranes, and scolices. Infection of the cyst causes fever and purulent sputum [1].

 In 1924, Drs. Alfredo Segers and Carlos Lagos Garcia from Argentina first described this sign in a 7-year-old girl with a pulmonary hydatid cyst [2]. Her radiograph demonstrated a floating crumpled membrane resembling leaves of the camalote, an aquatic plant native to the Amazon River basin (Figure 1C). Later authors have described this finding in ultrasound and magnetic resonance imaging of hydatid cysts in the liver, muscle, and other organs, naming it the water lily sign [1].

Jerray et al. describe the camalote sign in the chest radiographs of 55 of 386 (14%) cases of symptomatic hydatid lung disease in Tunisia [3]. Of 176 Turkish cases presenting with perforated pulmonary hydatid cysts, the camalote sign was the most common radiologic finding, occurring in 20% of cases [4]. Associated symptoms, such as cough, dyspnea, and hemoptysis, are nonspecific, making the sign of the camalote an important diagnostic clue for hydatid cyst rupture. Reported complications associated with the camalote sign include massive hemoptysis, acute respiratory failure, secondary echinococcosis, pneumonia, and, rarely, anaphylactic shock [5]. The triad of epidemiologic risk, hemoptysis, and the sign of the camalote is indicative of ruptured pulmonary hydatid cyst.
